# Supplementary material for: Are carbon emissions trading and green financial instruments synergistic? -Comprehensive quantitative research based on content analysis
Source: PLoS One. 2024 Mar 7;19(3):e0298601. doi: 10.1371/journal.pone.0298601 (PMC10919601; doi:10.1371/journal.pone.0298601)
Supplement: S1 Data — (ZIP) [file pone.0298601.s001.zip › Data/地级市Z_F工作报告.zip!/╣π╬≈/╬α╓▌╩╨/└┤╫╘╣1⁄2╓┌║┼í╛┬φ┐╦╩2╛▌═°í┐_2009.docx]

各位代表：

　　现在，我代表市人民政府向大会作政府工作报告，请予审议，并请市政协委员和其他列席会议的同志提出意见。

　　2008年工作回顾

　　2008年是大事多、喜事多、难事多的一年，也是我市应对新挑战、取得新成果、实现新突破的一年。在自治区党委、政府和市委的正确领导下，在市人大的有力监督和市政协的大力支持下，我们团结和依靠全市各族人民，坚持以邓小平理论和“三个代表”重要思想为指导，深入贯彻落实科学发展观，战胜各种自然灾害，沉着应对国际金融危机，解放思想，扎实工作，全力实施“科学发展三年计划”，经济社会发展取得了明显成效，全面完成了市十二届人大四次会议确定的各项目标任务。

　　经济实力进一步增强。全市实现地区生产总值400.1亿元，增长14.9%，万元地区生产总值能耗降低1.7%；全社会固定资产投资完成198.3亿元，增长31%；财政收入32.4亿元，增长19.9%；城镇居民人均可支配收入13268元，增长16.8%；农民人均纯收入3854元，增长18.5%。在国内外经济下滑的情况下，我市地区生产总值依然保持两位数增长，增幅高于全区平均水平，总量在全区排位由2007年的第7位上升到第6位，财政收入由第8位上升到第7位。

　　工业发展进一步加快。全年完成工业总产值582亿元，当年增量达150亿元；完成工业增加值191亿元。规模以上工业企业389家，增加44家，完成工业总产值384.4亿元，实现利润10.3亿元；产值超亿元企业87家，增加24家。市区37家重点工业企业完成工业总产值65.7亿元，实现利润4.5亿元，占全市规模以上工业企业利润的43.5%。六大优势产业共完成工业总产值219亿元，占规模以上工业总产值的57%。完成108万平方米工业厂房建设任务。岑溪、藤县、万秀、蝶山、长洲工业集中区通过自治区A类产业园区评审，全市现有5个A类产业园区，1个B类产业园区，2个自治区级工业园区，各类园区共完成工业总产值96亿元，增长23.4%，完成税收3.5亿元，增长35.6%。

　　农业基础进一步夯实。落实各项支农、惠农政策，发放水稻良种补贴等5项直补资金超过1.3亿元，是我市历史上支农力度最大、惠农补贴最多的一年。全年粮食总产量81.1万吨，增长3.6%，超额完成自治区下达的计划任务，粮食储备和供应充足，确保了粮食安全。大力发展桑蚕、木薯、六堡茶、砂糖桔、迟熟荔枝和水产养殖等农业特色产业，六堡茶已成为我市一个特色品牌；以砂糖桔为重点的亚热带名优水果生产发展迅速，全年水果总产量27.1万吨，增长59.5%；肉兔、蜂蜜的生产规模和加工水平居全区首位。农业产业化发展进一步加快，国家级、自治区级和市级农业产业化龙头企业达26家。农业标准化生产继续推进，全市有142.6万亩农产品种植基地通过自治区无公害、绿色、有机农产品生产基地认证，9个砂糖桔生产基地通过产品出口基地认证，33个养殖基地通过自治区无公害标准化生产认证，农产品质量安全水平进一步提高。以水利为重点的农村基础设施建设力度不断加大，完成投资8.9亿元。植树造林24.8万亩，其中速丰林24万亩，分别是自治区下达任务的135%和162%。全市抽调2823名干部下乡进村指导新农村建设，取得了明显成效。第一产业增加值增长4.1%，实现了大灾之年农业增产、农民增收、农村稳定。

　　交通建设进一步加强。全年完成投资43.5亿元，交通基础设施建设步伐加快。梧州至南宁高速公路全线贯通；马江至梧州、岑溪至筋竹高速公路加快推进；梧州至贵港、岑溪至水汶高速公路前期工作基本完成。县乡路网和农村公路建设进展顺利。洛湛铁路梧州段已完成铺轨，南广铁路梧州段开工建设。梧州港龙圩作业区1号泊位基本建成；赤水圩作业区进港一级公路和5号泊位建设加快；贵港至梧州二级航道整治推进顺利，西江“亿吨黄金水道”建设迈出新步伐。恢复开通梧州至广州、深圳两条航线。拆除鸳江大桥和桂江二桥两个收费站，搬迁莲花大桥收费站。

　　开放合作进一步扩大。成功举办“2008（梧州）西江经济（港航）发展论坛”，与南宁、贵港、云浮、肇庆、佛山五市签署了《西江经济（港航）发展论坛梧州共识》；抓住建设西江“亿吨黄金水道”上升为自治区发展战略的机遇，积极推进和参与西江流域经济开放开发，共谋区域经济发展。积极与贵港、玉林、贺州共同推进“广西东部承接产业转移协作区”建设，并已得到自治区的高度重视和支持。成功举办了第五届梧州国际宝石节，进一步推动宝石贸易繁荣和宝石深加工发展。招商引资成效显著，全年引进市外境内资金119.8亿元，增长37.8%，实际利用外资1.5亿美元，增长8.7%；梧州口岸进出口货物总值4.6亿美元，市属外贸出口总额3.3亿美元，增长15.9%。

　　人民生活进一步改善。全年投入改善和保障民生资金26.6亿元。城镇居民人均可支配收入、农民人均纯收入分别比上年增长16.8%和18.5%，其中农民增收是近几年最快的一年。全年城镇新增就业1.7万人，城镇下岗失业人员实现再就业5766人，城镇登记失业率为3.9%；完成农村劳动力转移培训4.8万人，实现转移就业4.6万人。养老保险覆盖面进一步扩大，基金征缴总量达8.6亿元。农房统保力度进一步加大，因灾倒塌民房重建任务全面完成。城乡低保标准得到提高，全市纳入城乡低保人数13.5万人，发放低保资金7307万元。城镇居民基本医疗保险全面启动，参保人数超过16万人；新农合工作全面铺开，参合农民达210万人，参合率达85.5%；全面建立城乡困难群众医疗救助制度。市政府为民办的12件实事全部完成，其中廉租房建设走在全区前列。

　　各项事业进一步发展。实施第四轮科技创新计划，全年科技项目资金投入2.3亿元。继续巩固提高“两基”工作，城乡义务教育阶段学生全部免除学杂费，农村义务教育阶段学生和城市低保家庭学生享受免费教科书，资助贫困生资金达9600万元；招收进城务工人员子女7458人接受城市义务教育，其中为7136名学生免除借读费。清理化解农村“普九”债务工作成效明显。教育设施不断完善，教育质量继续提高，本专科上线人数和上线率再创历史新高。中职招生1.7万人，超额完成自治区下达的招生计划；梧州职业学院建成并开始招生。广泛开展“广场文化”、“送戏下乡”活动，群众文化娱乐活动日益丰富。全年接待旅游人数、旅游总收入均有较大增长。藤县石表山景区被评为国家4A级旅游景区。不断完善公共卫生体系和基本医疗服务体系。深入开展爱国卫生运动，荣获自治区卫生城市称号。人口与计划生育工作水平进一步提高，人口自然增长率控制在10％。以内。市县两级首次联合举办市运会获得成功。积极推进节能减排工作，全面加强城市环境污染源的整治，关停高耗能、高污染企业30家，化学需氧量、二氧化硫排放量分别削减30.2%和4.1%，城市环境质量进一步改善，全年市区空气质量有效监测天数362天，空气质量优良率达100%。全市城镇污水和生活垃圾处理设施建设加快推进，市区第一污水处理厂建成投入使用，第二污水处理厂动工建设。注重政府自身建设，职能部门各项工作取得了新进步。中直、区直驻梧单位为我市经济社会发展作出了积极贡献，驻梧部队在抗洪救灾、突发事件处置等方面发挥了重要作用。工会、青少年、妇女儿童、老龄、残疾人等各项事业稳步发展。

　　社会稳定进一步巩固。全力推进“平安梧州”建设，社会治安综合治理和平安创建活动取得新成效。认真做好信访和人民调解工作，深入开展“大接访、大下访、大调解”活动，集中解决了一批群众反映强烈的突出问题。大力加强安全生产工作，深入开展安全隐患专项整治，加大产品质量和食品药品安全监管力度，有效遏制了重特大事故的发生。在北京奥运会、中国—东盟博览会、自治区成立50周年大庆等重大活动期间，做到“大事不出，中事不出，小事少出”，得到自治区党委、政府的高度肯定。国防教育、民兵预备役建设和双拥工作取得新成绩。切实加强民主法制建设。积极推进政务、村务、厂务公开。圆满完成村委会、居委会的换届工作。

　　各位代表，过去的一年我市实现经济平稳较快发展、社会大局和谐稳定、人民群众安居乐业，这是自治区党委、政府和市委正确领导的结果，是市人大和市政协监督与支持的结果，是全市各族人民团结拼搏、艰苦奋斗的结果。在此，我代表市人民政府，向全市广大干部群众，各位人大代表、政协委员，各民主党派、工商联、人民团体和社会各界人士，中直、区直驻梧单位，以及驻梧部队官兵，致以崇高的敬意和衷心的感谢！

　　在看到成绩的同时，我们也清醒地认识到前进中面临的矛盾和困难，工作中存在的问题和不足：由于国际金融危机持续蔓延等多种原因，我市经济增速出现回落，部分企业生产困难、效益下滑，经济运行中的一些突出问题和深层次矛盾仍有待解决；经济总量不大，产业结构不尽合理的问题依然突出；农业产业化水平还不高，农民持续增收的渠道不多，统筹城乡发展困难仍然较大；就业压力增大，安全生产形势还比较严峻；社会事业发展还存在薄弱环节，一些公共服务设施建设还相对滞后；政府工作与人民的期望还有一定差距，机关干部的忧患意识、创新意识、服务意识仍有待增强。我们一定要重视这些困难和问题，以对党、对人民高度负责的使命感和紧迫感，更加沉着应对，更加努力工作，决不辜负人民的重托。

　　2009年工作目标

　　2009年将是新世纪以来我市经济社会发展面临困难与挑战最为严峻的一年。国际金融危机导致国际、国内经济环境恶化，风险和不确定性因素增大，我们将面临前所未有的挑战：外向型经济增长乏力，产业转移步伐放缓，部分企业生产经营陷入困境，效益大幅下滑，农民增收难度加大，社会就业形势严峻，经济增长下行的压力明显。

　　面对压力和挑战，我们更要充分认识危机中蕴含的重大发展机遇和积极因素：一是国家大规模增加政府支出和实施结构性减税的政策措施正在发挥积极作用，自治区确定6000亿元的年度投资规模和建设西江“亿吨黄金水道”的战略，有利于促进我市保持较大的投资规模，有利于推进企业自主创新和产业升级，有利于加快港航基础设施和综合交通网络建设，对我市的经济发展将起到积极的拉动作用。二是通过近几年的努力，我市综合交通体系日趋完善，投资环境日趋优化，经济实力日趋增强，一批重大基础设施和产业项目将陆续开工建设或建成投产，具备了进一步加快发展的条件。

　　“信心就是力量”。我们既要充分估计和清醒认识前进中的困难和问题，又要充分认识和善于把握难得的发展机遇和有利条件，增强战胜困难、迎接挑战的信心和勇气，化压力为动力、化危机为转机、化被动为主动，以超常规的发展思维、超常规的发展方式、超常规的保证措施、超常规的拼搏精神，克难攻坚，努力把金融危机带来的影响减少到最低程度，继续开创梧州经济社会持续平稳较快发展的新局面。

　　2009年政府工作的总体要求是：认真贯彻落实党的十七大、十七届三中全会、中央和自治区经济工作会议以及自治区十一届人大二次会议精神，全面贯彻落实科学发展观，按照自治区党委、政府和市委的统一部署，以保增长、保民生、保稳定为首要任务，开展“项目攻坚年”和“服务企业年”活动，全力推进交通基础设施建设，全力推进重大产业和园区建设，全力推进农业基础设施建设，全力推进城镇基础设施建设，全力推进民生工程，确保经济社会又好又快发展，加快把我市建设成为区域性枢纽城市和承接产业转移示范基地，以优异成绩迎接新中国成立60周年。

　　经济社会发展的主要预期目标为：地区生产总值增长13%，力争15%；财政收入增长11%，力争15%；工业增加值增长17%，力争22%；固定资产投资增长35%，力争60%；市属外贸出口总额增长10%，利用外资增长6%，引进市外境内资金增长21%，全社会消费品零售总额增长15%，城镇居民人均可支配收入增长10%，农民人均纯收入增长10%，城镇登记失业率控制在4.5%以内，人口自然增长率控制在10％。以内；万元地区生产总值能耗下降3.8%，主要污染物排放总量控制在自治区下达的指标以内。

　　以上目标，与我市“十一五”规划和“科学发展三年计划”的要求相衔接，符合科学发展的要求，符合中央、自治区的战略部署，有利于增强信心、稳定预期。在不确定性因素增加的形势下，实现经济增长13%难度较大，必须经过全市上下共同努力才能实现。为此，我们必须把应对国际金融危机当作加强基础设施建设的重大机遇、推动产业结构优化升级的重大机遇、缩小与发达地区和区内先进城市差距的重大机遇，以非常办法、非常措施、非常力度、非常政策，谋求大战略，启动大规划，开展大建设，实现大发展。

　　谋求大战略——就是要推动建设西江“亿吨黄金水道”战略上升为两广省区战略，力争上升为国家战略；推动桂东四市承接产业转移基础设施建设大会战上升为自治区战略；推动进口再生资源加工园区上升为国家级循环经济示范区，构建国家级再生资源循环加工利用基地的有效载体；规划设立梧州出口加工区，努力把西江“亿吨黄金水道”和承接产业转移示范基地打造成为促进梧州加快发展的“两大平台”，促进梧州经济腾飞的“一对翅膀”。

　　启动大规划——就是要围绕建设区域性枢纽城市和承接产业转移示范基地的城市定位，高起点、高标准启动城市发展规划，进行城市发展战略方向研究，开展统筹城乡发展规划、重大产业发展规划、综合交通体系发展规划、生态环境保护规划和其他相关配套的专项规划，形成较为完善的城市发展规划体系，以大规划引领大发展。

　　开展大建设——就是要在确保农业稳定发展和社会大局稳定的基础上，抓住项目建设这个关键，掀起项目建设新高潮，实现交通基础设施、重大产业发展和城镇建设的新突破，实现经济持续平稳较快发展，不断改善民生，让百姓享受更多的发展成果。

　　实现大发展——就是要按照“三年打基础、五年大发展”的战略部署，以资源换产业、以存量换增量、以时间换空间，全力打造区域性加工制造基地、物流基地、商贸基地和旅游休闲基地，确立梧州作为区域性枢纽城市和承接产业转移示范基地的地位。

　　各位代表，谋求大战略，启动大规划，开展大建设，实现大发展，是贯彻落实中央和自治区重大部署，保增长、保民生、保稳定的重要举措。我们必须横下一条心，不动摇、不懈怠、不折腾，采取最直接、最有力、最有效的措施，努力实现我市的科学发展、和谐发展、跨越发展。

　　2009年工作措施

　　为实现今年经济社会发展的各项目标任务，必须围绕中央、自治区和市委的重大决策，以新思路谋求新发展，以新举措赢得新突破。主要抓好以下10项工作：

　　一、以推进重大项目为重点，不断增强投资的拉动力

　　坚持投资拉动经济的战略，把项目建设作为经济工作的重中之重，全年安排总投资超1000万元的重点项目329个，年内计划投资198亿元，力争完成235亿元，全社会固定资产投资总额力争达到320亿元。

　　千方百计推动项目开工。采取超常规办法，努力把今后3年要开工的项目集中到今年开工建设，计划新开工项目158个，年内计划投资72亿元，力争完成83亿元。其中：交通项目14个，年内计划投资17亿元，力争完成21亿元，开工建设梧州至贵港、岑溪至水汶高速公路、长洲水利枢纽3线、4线船闸等项目。能源项目13个，年内计划投资5亿元，开工建设城市电网改造、输变电工程等项目。城市基础设施项目36个，年内计划投资11亿元，力争完成14亿元，开工建设地质灾害整治、平民冲人居环境整治和藤县、蒙山污水处理厂等项目。农林水项目6个，年内计划投资1亿元，力争完成2亿元，开工建设市区河东防洪堤桂江段加固工程、农村户用沼气等项目。工业项目57个，年内计划投资30亿元，力争完成32亿元，开工建设林浆纸一体化、木薯燃料乙醇、中冶工业园等项目。社会事业项目16个，年内计划投资4亿元，力争完成5亿元，开工建设市文化中心改造、市职业教育中心、市福利院老年人公寓等项目。商贸流通项目16个，年内计划投资4亿元，开工建设义乌小商品城、烟草物流中心等项目。

　　千方百计推进在建项目。加快171个续建项目建设进度，尽可能多地形成投资工作量，年内计划投资126亿元，力争完成152亿元。其中：交通项目9个，年内计划投资37亿元，力争完成50亿元，主要建设南广铁路、洛湛铁路梧州段，马江至梧州、岑溪至筋竹高速公路，贵港至梧州二级航道整治等项目。能源项目7个，年内计划投资16亿元，主要建设长洲水利枢纽、旺村水利枢纽、蝶山输变电工程等项目。城市基础设施项目42个，年内计划投资26亿元，力争完成29亿元，主要建设红岭新区路网、西江三桥和岑溪、苍梧污水处理厂等项目。农林水项目9个，年内计划投资3亿元，力争完成4亿元，主要建设长洲防洪堤南北堤扩建、农村饮水安全等项目。工业项目84个，年内计划投资38亿元，力争完成45亿元，主要建设进口再生资源加工园区一期、新中陶陶瓷、五一塑料公司和天鹅蓄电池公司搬迁技改等项目。社会事业项目13个，年内计划投资3亿元，力争完成4亿元，主要建设梧州职业学院一期、市红会医院扩建等项目。商贸流通项目7个，年内计划投资3亿元，力争完成4亿元，主要建设六堡茶专业市场和易达物流中心、同舟农产品物流配送中心等项目。

　　千方百计突破项目前期工作。加大项目前期工作经费投入，市本级今年安排2000万元，各县（市、区）分别安排500万元，确保项目前期工作高效有序推进。重点突破梧州火电、西气东输二线工程梧州段、环城高速公路等41个重大项目的前期工作，形成“完工一批、开工一批、前期突破一批、招商引进一批”的项目推进机制，实现项目建设的良性循环。

　　千方百计落实项目建设资金。全力争取国家和自治区的支持，积极组织项目申报，切实加强与上级有关部门的衔接、沟通，争取更多的项目列入国家和自治区计划盘子。全面加强政银企合作，扩大银行信贷规模，力争新增信贷投放量100亿元。发挥好财政资金的引导作用，鼓励民间资金投入各类项目建设，进一步整合政府资金，集中财力办大事。发挥东泰公司等融资平台作用，力争新增融资56亿元。积极引进国有大企业、大集团和战略投资者，推进神冠蛋白肠衣公司、日成林化公司、华虹丝绸公司等企业上市或发行企业债券。支持中恒集团、索芙特公司在资本市场上开展再融资工作，不断增强我市骨干企业的融资能力，进一步扩大直接融资规模。

　　千方百计确保项目质量。完善项目建设的指挥系统、组织系统和监督系统，建立项目联合审批工作机制、项目工作目标责任制和奖惩制度，集中组织、集中审批、集中办理、集中征地拆迁。依法依规推进项目工作，既要采取超常规方式，也要按法定程序完善各项手续。监察、审计等部门要全面介入项目建设，实施全程监督和检查，确保建成“优质、安全、廉政”的工程。

　　二、以培育支柱产业为重点，不断增强工业的扩张力

　　做优产业。加大力度支持六大优势产业和三大特色产业发展，力争机械制造产业率先实现产值超100亿元。鼓励引导造船企业以及有色金属、松香、木材、钛白粉、高岭土、医药、食品、桑蚕等资源型企业兼并、重组和联合，推动产业规模有效扩张，把我市的资源优势真正转化为产业优势。

　　做强企业。加大资源整合力度，鼓励骨干企业建立原料生产基地，进一步集中优势资源，扶持中恒集团、神冠蛋白、三威林产、日成林化等一批骨干企业做强、做大、做优。抓好重点技改项目，力争全年完成技改投资66亿元。加快以企业为主体、产学研相结合的技术创新体系建设，力争国家级高新技术企业达到5家以上。支持符合产业发展要求的中小企业借力发展、投大靠强、优化重组、转型升级。新增年产值超亿元的企业16家、超5亿元的企业8家、超10亿元的企业1家。培育年纳税超1000万元的企业29家、超5000万元的企业7家、超亿元的企业4家。

　　做大园区。大力争取自治区资金投入，进一步加大园区基础设施建设力度。积极引进产业关联度高、财税贡献大、科技含量高的项目，同时加大配套企业招商力度，提高产业集聚度，形成各具特色的产业园区。力争全市各园区完成基础设施投资14.5亿元，完成工业总产值115.3亿元，实现税收4.7亿元。其中梧州工业园区完成投资8000万元，完成工业总产值20亿元，实现税收1.2亿元；苍梧工业园区完成投资2.4亿元，完成工业总产值23.5亿元，实现税收6000万元；进口再生资源加工园区完成投资4.5亿元，完成工业总产值9.9亿元，实现税收1500万元。

　　做好服务。坚持“一企一策”，建立健全对重点产业、重点企业的服务机制，通过财政支持、技改贴息、专项补贴和奖励、探索直供电等方式，扶持重点产业、重点企业尤其是纳税超100万元的企业发展。加快融资担保体系建设，切实解决中小企业融资难问题。进一步清理涉企行政事业性收费，严肃查处“三乱”行为，杜绝不切实际的检查评比。加强经济运行监测，对可能出现的不确定情况作出快速反应，帮助企业解决实际困难。

　　三、以促进农民增收为重点，不断增强农业的支撑力

　　确保粮食安全。全面落实粮食安全行政首长责任制和粮食生产扶持、补助政策，充分调动农民的种粮积极性，稳定播种面积，大力推广高产优质的超级稻、杂交水稻组合以及增产增收技术措施，提高粮食单产，增强粮食综合生产能力，确保全市粮食播种面积稳定在236万亩左右、总产量达82万吨以上，保障粮食储备充足和市场供应平稳。

　　大力发展特色产业。建立工业化与农业产业化良性互动机制，将农业特色产业基地与工业原料基地建设相结合，推进农业产业化。抓好六堡茶的良种茶苗繁育、原料茶基地建设，提升加工工艺，培育壮大六堡茶产业。加快玉桂八角香料产业发展，积极开发香料深加工。加快水产畜牧业发展，大力开发长洲水利枢纽库区淡水养殖。扶持农业产业化龙头企业发展，发挥龙头企业和专业合作社带动基地建设的作用，建设一批专业村专业镇。引导农村土地使用权合理流转，促进优势产业向区域化、专业化、规模化发展。抓好珠江防护林、退耕还林等国家重点工程项目，落实退耕还林和生态公益林补助政策，完成造林13.6万亩。开展集体林权制度改革试点，为全面推进林权制度改革探索经验。

　　加快以水利为重点的农村基础设施建设。大力开展农村水利设施建设，计划投资2.7亿元，实施水利建设项目234个。其中：维修加固病险水库27座，实施农村饮水安全工程71处，修复水毁水利工程25处，完成渠道清淤2154公里，新增或恢复灌溉面积1万亩以上，改善灌溉面积2万亩以上。继续推进新农村建设。

　　完善农村社会化服务体系。继续深化和拓展科技特派员制度，培育多元化和专业化社会服务组织。加快建立新型农技推广服务网络，完善重大动植物检疫防控、农产品质量安全、农村信息等服务体系，重点建设新一代雷达信息处理中心、兽药产品质量检测中心以及覆盖全部乡镇的动物防疫冷链设施。全面落实农机购置补贴政策，加快实施“千乡万村现代农机装备推进工程”。鼓励金融机构大力发展农村小额信贷，推广利农便农的金穗惠农卡等金融服务。

　　大力发展县域经济。以农民增收、财政增长、扩大就业为目标，统筹城乡发展，促进产业合理布局，把农产品加工业培育成为县域经济的特色产业。以园区为载体，加快承接产业转移步伐，吸引劳动密集型、资源加工型产业向县域集聚。积极发展小城镇经济，以产业聚集带动小城镇发展，使小城镇成为县域经济发展的平台和增长点。

　　四、以建设港口码头为重点，不断增强交通的带动力

　　加快港口码头建设。加快推进梧州港赤水圩作业区、龙圩作业区和李家庄码头三期的建设，争取赤水圩作业区建成2000吨级泊位5个、龙圩作业区建成1000吨级泊位3个。积极做好藤县河东作业区、桂江大漓口作业区的前期工作。加快贵港至梧州二级航道整治，实现2000吨级船舶全线通航。加快赤水圩作业区进港一级公路和进港铁路支线建设，推进龙圩至再生资源加工园区、龙圩至塘步一级公路建设，实现港口码头与高速公路、铁路站场、工业园区有效连接，推进临港经济发展。

　　加快铁路项目建设。做好南广铁路梧州段征地拆迁工作，密切加强与铁路部门的协作与沟通，为项目建设创造良好环境，促进项目建设快速推进。配合支持铁路部门加快洛湛铁路梧州段建设，按时完成火车站场和站前大道建设任务。争取开工建设柳州至肇庆铁路梧州段和岑溪至罗定铁路岑溪段。

　　加快公路项目建设。做好梧州至贵港、岑溪至水汶高速公路的征地拆迁工作。建成潭东至太平二级公路，开工建设苍梧大坡至广平、藤县同心至岑溪三堡等13条联网公路。加快建设龙船冲客运中心、金晖汽运中心扩建工程，确保年内投入使用。加快推进长洲龙华至藤县津北、倒水至平浪联网公路等项目前期工作。

　　加快推进机场扩建。积极争取自治区支持，开工建设机场航站区和飞行区改造工程，进一步完善机场基础设施，提高综合配套服务功能，大力开辟新的航线航班，努力把梧州机场建成桂东航空运输枢纽。

　　五、以承接产业转移为重点，不断增强开放合作的推动力

　　加大招商引资力度。坚持以粤港澳台为招商主战场，确立“靠大引强”招商思路，围绕新建工业厂房，加强与世界500强、国内500强特别是中央企业的联系与沟通，努力引进产业关联度大、技术水平高的大企业、大项目。加强项目跟踪和落地服务，推进招商引资大兑现工作，提高项目的合同履约率、资金到位率和开竣工率，力争招商引资实际到位资金156亿元，其中利用外资1.59亿美元。

　　加强区域经济合作。积极参与中国—东盟自由贸易区、珠三角经济区、北部湾经济区等区域性经济合作，在合作中实现产业对接、项目对接。加强西江沿岸城市合作，建立经济合作机制，促进西江经济带快速发展。推动与玉林、贵港、贺州加快建立承接产业转移协作区，争取自治区在资金项目上给予更多支持。继续办好梧州国际宝石节，推动经济贸易，扩大经济合作。

　　大力优化投资环境。积极推进梧州口岸赤水圩作业区列入国家口岸开放规划，规划建设广西电子口岸梧州物流服务平台和梧州口岸区域物流中心，开通往来港澳陆路直通车业务，建立梧州进出境快件中心。推行海关便捷通关措施，简化报检手续，提高口岸通关效能。深化行政审批制度改革，最大限度地放宽投资领域，简化审批手续，减少审批环节，规范收费事项，降低投资成本。推进政务中心“一门受理，并联审批”的工作方式，提高办事效率。切实解决外来投资者反映的突出问题，保护投资者合法权益。

　　六、以建设“五大工程”为重点，不断增强城市的竞争力

　　实施中心城区“五大工程”。（1）新区拓展工程。加快红岭片、三龙片开发步伐，推进行政区划调整，启动南岸片基础设施建设前期工作，力争城市新区开发取得大的突破，进一步拉开城市框架，扩大城市建成区面积。（2）城市绿色工程。加强城市主次干道、城乡结合部绿化工作，重点抓好珠山、榜山、白云山景区绿化工程。加大城市公共绿地建设力度，实施拆墙透绿、庭院植绿、沿街布绿，提高城市绿化覆盖率。（3）城市美化工程。加大“城乡清洁工程”力度，加强城市主风格、主色调的规划建设，对西堤路、新兴路主干线和龙圩至西江大桥道路沿线，以及市区其他道路沿街建筑进行“穿衣戴帽”，优化市区灯光工程，形成统一协调的“城市景观主题”，提高城市品位。（4）环境保护工程。加大节能减排工作力度，加快建设污水处理以及生活垃圾无害化处理工程。加强环境监管，加大污染治理力度，改善空气质量。积极推进生态市建设步伐，加快生态县乡和生态小区建设，保护“母亲河”西江的生态环境，使水更清、山更绿、城更美。（5）城市创优工程。加强城市管理，优化城市环境。开展全民创优活动，使城市交通、环境、卫生、治安等各方面明显改善，实现城乡富裕文明、生活便捷舒适、社会和谐稳定，努力创建国家历史文化名城、国家森林城市、国家园林城市，打造宜居城市。

　　加快县城和重点镇建设。继续完善县域总体规划、乡镇和中心村规划修编，优化城乡空间布局。岑溪要实现率先发展，加快建设成为中等城市。苍梧县城要充分发挥紧靠中心城市和交通要道的优势，建设成为梧州市的物流中心和新兴工业基地。藤县县城要抓住铁路、高速公路、西江“亿吨黄金水道”、新港码头以及林浆纸一体化项目建设等机遇，加快向中等城市迈进的步伐。蒙山县要依托桂林旅游圈和良好的自然条件，发挥旅游和绿色产业优势，继续推进县城基础设施建设，扩大城区规模。依托交通干线、旅游通道，加快石桥、新地、塘步、太平、归义、黄村等重点镇发展，构建中心城市、副中心城市、县城中心城镇、重点镇和一般集镇大小结合、梯次分布、功能互补、特色鲜明的城镇群、城镇带，实现以城带乡、以工促农，推动城乡互动发展，提高城镇化水平。开展以主要公路沿线、沿江、景区景点周边、城乡结合部为重点的镇容镇貌、村容村貌整治，改善城乡居住环境。

　　七、以发展商贸物流为重点，不断增强现代服务业的促进力

　　大力发展商贸物流业。加快港口物流业发展，规划建设龙圩、赤水物流园区，通过专业化、规模化、市场化运作，对储存、运输、配送、销售等资源进行优化配置，进一步推动现代物流业发展，增强枢纽城市的聚集和辐射功能。规划建设红岭新区商业中心、梧州工业园区商贸中心，加大汽车、机械、电子、建材等专业市场培育力度。继续推进“万村千乡市场工程”和“双百市场工程”，积极做好“家电下乡”工作，大力促进农民消费、农产品流通。

　　加快旅游休闲基地建设。加大旅游资源整合和开发力度，深入挖掘千年古城文化和百年商埠文化，加大基础设施投入，加强景区景点建设，打造特色鲜明、具有区域影响力的旅游品牌。加强与桂林、南宁、广州、深圳等地有实力的旅游企业合作，牢牢抓住珠三角，积极拓展区内和港澳台以及东盟国家的旅游客源市场，推动休闲度假业加快发展。

　　加快新型服务业发展。推进投融资体制改革、金融组团及金融服务创新，鼓励和扶持各类非银行金融机构的发展。大力发展知识密集型中介服务机构，借鉴和吸纳国内外新的服务品种、方式和经营理念，推动中介服务行业的改造和升级。创新社区服务模式，积极拓展社区养老、卫生、教育等服务项目，提升服务水平。加快发展宾馆休闲餐饮娱乐等服务产业，促进消费增长。

　　稳定房地产市场发展。加强住房建设规划，调整和优化住房供应结构，加快发展二手房市场和住房租赁市场。落实鼓励普通商品住房消费的财税、信贷政策，减轻居民购买普通商品住房负担。积极推进廉租住房建设，通过政府新建、收购、改建和鼓励社会捐赠等方式增加廉租住房供应。加大房地产市场监管力度，维护房地产市场秩序。

　　八、以改善民生为重点，不断增强和谐社会的凝聚力

　　进一步扩大就业。全面落实就业促进法和鼓励创业的税收优惠、小额担保贷款、资金补贴等各项政策措施，统筹推进城镇新增劳动力、大中专毕业生、城镇登记失业人员、返乡农民工和农村劳动力转移就业工作，力争全市城镇新增就业1.4万人、城镇下岗失业人员实现再就业4000人，城镇登记失业率控制在4.5%以内。加大就业服务投入，完善公共就业服务体系，进一步强化农村劳动力转移就业的指导和服务。加强创业培训和职业技能培训，提高劳动者创业能力和创业水平。

　　进一步完善社会保障体系。加强社会保险基金征缴工作，着重抓好非公企业、灵活就业人员、农民工和大学生的社会保险扩面工作。加快推进新型农村合作医疗保险和城镇居民基本医疗保险工作。稳步推进失业保险市级统筹工作，建立健全失业保险预警制度。进一步完善城乡低保和医疗救助制度，为城乡特困群众和弱势群体提供基本生活和医疗保障。做好农民工的社会保障和公共服务工作，合理贯彻落实最低工资标准。努力实现老百姓老有所养、病有所医、困有所助，生活更有保障。

　　加大扶贫开发力度。加大扶贫投入，实现农村未解决温饱人口减少2.1万人以上。大力实施扶贫开发项目，启动第三批27个贫困村的整村推进扶贫，稳定解决贫困村的温饱问题。继续抓好长洲和旺村水利枢纽库区移民工作，维护移民群众合法权益。落实好国家大中型水库移民后期扶持政策，逐步改善库区移民的生产生活条件。

　　九、切实为民办实事，让群众得到更多实惠

　　今年继续从解决群众最关心、最直接、最紧迫的问题入手，投入资金8.7亿元，全力办好以下12件实事：

　　（一）社会保障惠民工程。（1）实施城镇居民基本医疗保险制度。筹措2500万元，实现参保人数达25万人以上。（2）支持返乡农民工就业创业。筹措2700万元，实施返乡农民工职业技能培训、鉴定，奖励自主创业人员或吸纳返乡农民工就业的用工单位。（3）完善新型农村合作医疗制度。筹措2.1亿元，标准由80元/人提高到100元/人，农民参合率达90%以上。（4）完善城乡居民最低生活保障制度。筹措1200万元，城市低保对象月人均补助水平由130元提高到150元，农村低保对象月人均补助水平由35元提高到45元。（5）实施乡乡镇镇有敬老院项目。筹措1190万元，其中各县（市、区）政府配套资金340万元，新建17个乡镇敬老院。

　　（二）教育惠民工程。（1）资助普通高中家庭经济困难学生。筹措150万元，资助1500人，每人每年1000元。（2）资助中职学生学费。筹措225万元，对进入中等职业学校学习的库区移民、扶贫异地安置家庭、城镇低保人员、农民特困家庭子女、退伍士兵等特定人员给予学费资助，资助1500人，其中农村学生1200元/人，城市学生1800元/人。（3）资助中职学生第三学年生活费。筹措75万元，对中等职业学校难以通过工学结合、顶岗实习获得稳定收入的特定专业学生继续给予第三学年生活费资助，资助500人，每人每年1500元。（4）资助家庭经济困难大学新生。筹措68万元，资助1500人，其中区内院校录取的贫困新生每人400元；长江以南区外院校录取的贫困新生每人500元，长江以北区外院校录取的贫困新生每人600元。

　　（三）乡村社会服务工程。（1）乡村文化基础设施建设及活动设备配置。筹措720万元，建成10个乡镇综合文化站，为29个乡镇综合文化站、75个村委会配置文化活动设备。（2）继续扩大“万村千乡市场”工程建设。新建农家店300家以上，其中农民生产资料店100家、日用品店200家。（3）家电下乡。筹措498.3万元，对农民购买彩电、冰箱、洗衣机和手机等中标家电产品给予资金补助。（4）基层医疗卫生服务能力建设。筹措1724万元，新建3个城市社区卫生服务中心和17个乡镇卫生院，加强30个乡镇卫生院能力建设。

　　（四）农村基础设施建设工程。（1）县乡公路建设。筹措2.43亿元，新增6个乡镇、105个建制村通等级硬化路。（2）村屯通广播电视。筹措1212万元，实施55个乡镇1010个20户以上的自然村通广播电视工程。（3）村村通电。筹措1659万元，实施全市1096户无电农村居民通电工程。（4）农村饮水安全工程。筹措3436万元，解决8.3万农村居民饮水安全问题。（5）农村沼气池工程。筹措2336万元，新建沼气池11680座，力争沼气池入户率达33%以上。

　　（五）保障性住房建设工程。筹措1.2亿元，开工建设20万平方米以上的保障性住房。

　　（六）市区路桥收费整合工程。继续整合苍梧环城一级公路、紫竹大桥、云龙大桥、西江大桥等市区路桥收费站，实现市区“河东河西、江南江北”行车无障碍，方便百姓出行。

　　（七）市民休闲公园建设工程。筹措3100万元，建设珠山六堡茶文化公园和榜山、河西老码头市民休闲公园。取消珠山、中山公园门票。

　　（八）县乡计生服务设施建设工程。筹措846万元，建设县级计生服务站1个和乡镇计生服务所18个，总建筑面积10250平方米。

　　（九）城市社区服务设施建设工程。筹措425万元，新建蝶山区东兴街道社区服务中心、下三云社区服务站和富民街道富康、榜山、富新社区服务站。

　　（十）市区地质灾害整治工程。筹措1000万元，继续整治市区地质灾害隐患，并对已整治的工程项目进行完善和维护。

　　（十一）农村贫困残疾人危房改造工程。筹措300万元，每户补助6000元，改造500户农村贫困残疾人的危房。

　　（十二）乡镇兽医站建设工程。筹措154万元，建设10个乡镇水产畜牧兽医站。

　　各位代表，为民办实事是政府的重要职责，我们一定全力把这些实事办快、办好、办实，让更多的群众得到更多的实惠！

　　十、坚持统筹兼顾，扎实推进各项社会事业全面发展

　　优先发展教育事业。全面实施素质教育，加强学校管理和教师队伍建设，加大中小学校舍建设力度，优化中小学校布局，合理配置教育资源，扎实推进城市义务教育阶段学生免除杂费和取消借读费工作，切实解决适龄儿童入学难问题，进一步巩固 “两基”成果和提高办学水平。大力实施中等职业教育攻坚，整合市、县两级职业教育资源，扩大职业教育办学规模。加快高等教育发展，办好梧州学院和梧州职业学院。

　　加快发展文化事业。加大文化事业投入，开工建设市图书馆、博物馆，加强县级图书馆、文化馆、乡镇文化站及农村广播电视覆盖等公益性项目建设。以打造“西江龙母”文化品牌为重点，推出一批反映时代精神和地方特色的艺术精品，推动文化产业的发展。以“璀璨广场”和“和谐文化进社区”为龙头，推进群众文化繁荣和发展。加强文化市场管理，加大文化遗产保护力度，完成第三次文物普查实地调查工作。抓好新闻出版、社科研究、地方志工作。

　　加强人口和计划生育工作。提高计划生育依法行政水平，严格控制政策外生育，稳定低生育水平。加强计生服务基础设施建设，推进人口计生体制机制创新，在苍梧县部分乡镇开展乡村卫生院（室）、计生服务站（室）整合试点工作。加强流动人口计划生育服务和管理。大力实施新农村新家庭计划，开展“婚育新风进万家” 活动和“关爱女孩行动”，综合治理出生人口性别比偏高问题，严厉打击“两非”行为。

　　加快发展卫生、体育等各项事业。加强卫生设施建设，提高医疗水平。全面推行新型农村合作医疗制度，加强城市社区医疗卫生服务体系建设。做好重大疾病防控工作。大力发展体育事业，开工建设市体育中心，办好市第27届青年运动会和全市“千村万户”运动会。继续推进第四轮科技创新计划活动，不断提高自主创新能力。加强各级各类人才队伍建设，推进“广西新世纪十百千人才工程”。全面落实侨务政策，依法保护归侨、侨眷合法权益。完善社会专项事务管理，加快发展慈善事业。做好民族、宗教、外事、对台、编制、统计、档案、气象、人防、地震以及残疾人、老龄等工作，充分发挥各民主党派、工商联以及工青妇等人民团体的桥梁纽带作用。切实加强国防和民兵预备役建设，提高民兵预备役执行任务能力。广泛开展国防教育，增强全民国防安全观念。深入开展双拥共建活动，增进军政军民团结。

　　加强安全生产和维护社会稳定。严格落实各级政府安全生产监管责任和企业安全生产主体责任，加大隐患的排查和治理力度，依法严厉查处各类安全生产违法违规行为，防范和遏制重特大安全事故。加强产品质量和食品药品安全监管，确保食品药品安全。开展法制宣传教育，加强基层人民调解建设。加强信访工作，深入开展“大接访”活动，着力解决突出的信访问题。高度重视经济领域以及重大项目建设、国有企业改制等可能影响社会稳定的突出问题，排查化解社会矛盾纠纷。建立健全自然灾害和公共安全突发事件监测预警体系和信息报告、应急救援、社会动员机制，有效预防和处置各类突发公共事件。加强社会治安综合治理，完善社会防控体系，深入开展禁毒工作，依法打击违法犯罪活动，切实维护社会稳定。

　　各位代表，严峻的形势、艰巨的任务对政府工作提出了更高的要求。必须加强政府自身改革和建设，增强贯彻落实科学发展观的自觉性和坚定性。进一步加强和改善经济调节和市场监管，更加注重履行社会管理和公共服务职能。进一步健全重大工作部署督办、机关绩效考评、行政效能监察等激励监督机制，推进政务公开，加强电子政务建设。坚决克服形式主义、官僚主义，坚决摆脱文山会海。坚持勤俭办一切事业，从严控制一般性支出，对公务购车用车、会议、公务接待、党政机关公务人员出国（境）等经费预算实行零增长。严格执行党风廉政建设责任制，加强审计监督，切实做到廉政勤政，坚决纠正损害群众利益的不正之风。始终牢记全心全意为人民服务的宗旨，真正做到“忧民所忧、求民所求、乐民所乐、安民所安”。

　　各位代表，今年经济社会发展任务艰巨而繁重，让我们紧密团结在以胡锦涛同志为总书记的党中央周围，高举中国特色社会主义伟大旗帜，深入贯彻落实科学发展观，抢抓发展机遇，广集各方智慧，坚定信心，迎接挑战，以非凡力度和超强干劲，开拓进取，扎实工作，全面完成今年各项目标任务，以优异成绩迎接新中国成立60周年！
